# Aberrant Innate Immune Activation following Tissue Injury Impairs Pancreatic Regeneration

**DOI:** 10.1371/journal.pone.0102125

**Published:** 2014-07-10

**Authors:** Alexandra E. Folias, Cristina Penaranda, Anthony L. Su, Jeffrey A. Bluestone, Matthias Hebrok

**Affiliations:** Diabetes Center, Department of Medicine, University of California San Francisco, San Francisco, California, United States of America; Garvan Institute of Medical Research, Australia

## Abstract

Normal tissue architecture is disrupted following injury, as resident tissue cells become damaged and immune cells are recruited to the site of injury. While injury and inflammation are critical to tissue remodeling, the inability to resolve this response can lead to the destructive complications of chronic inflammation. In the pancreas, acinar cells of the exocrine compartment respond to injury by transiently adopting characteristics of progenitor cells present during embryonic development. This process of de-differentiation creates a window where a mature and stable cell gains flexibility and is potentially permissive to changes in cellular fate. How de-differentiation can turn an acinar cell into another cell type (such as a pancreatic β-cell), or a cell with cancerous potential (as in cases of deregulated Kras activity) is of interest to both the regenerative medicine and cancer communities. While it is known that inflammation and acinar de-differentiation increase following pancreatic injury, it remains unclear which immune cells are involved in this process. We used a combination of genetically modified mice, immunological blockade and cellular characterization to identify the immune cells that impact pancreatic regeneration in an *in vivo* model of pancreatitis. We identified the innate inflammatory response of macrophages and neutrophils as regulators of pancreatic regeneration. Under normal conditions, mild innate inflammation prompts a transient de-differentiation of acinar cells that readily dissipates to allow normal regeneration. However, non-resolving inflammation developed when elevated pancreatic levels of neutrophils producing interferon-γ increased iNOS levels and the pro-inflammatory response of macrophages. Pancreatic injury improved following *in vivo* macrophage depletion, iNOS inhibition as well as suppression of iNOS levels in macrophages via interferon-γ blockade, supporting the impairment in regeneration and the development of chronic inflammation arises from aberrant activation of the innate inflammatory response. Collectively these studies identify targetable inflammatory factors that can be used to influence the development of non-resolving inflammation and pancreatic regeneration following injury.

## Introduction

Tissue injury is a destructive process that creates cellular disorganization and an influx of immunological cells and factors [1], [2]. The inflammation generated in response to injury can increase cellular flexibility and promote tissue renewal by influencing tissue outgrowth, branching, organization and remodeling [2]–[6]. However, failure to resolve acute inflammation following injury can lead to the development of the chronic inflammation that contributes to the pathogenesis of several diseases [4], [7], [8]. Understanding the impact immune cells have on tissue cells during injury-resolution can advance the design of an integrated therapeutic approach aimed at resolving inflammation in either a universal or tissue-specific manner.

The pancreas is highly susceptible to inflammation and pancreatitis [9]–[11], and injury is known to drive pancreatic acinar cells to de-differentiate into cells reminiscent of progenitor cells present during embryonic development [10], [11]. During this de-differentiation process, acinar cells undergo substantial morphological and molecular changes as they assume a more duct-like state [11], [12]. In the absence of aberrant signaling, this ductal transition is transient and the cell will revert back to an acinar fate [10]–[14]. However, in cases of chronic injury or deregulated oncogenic Kras activity, this ductal progenitor-like state can be stabilized [15]. Chronic pancreatitis is a potent risk factor for the development of pancreatic ductal adenocarcinoma (PDAC) in humans [15]–[18], and several mouse models have demonstrated injury and inflammation as permissive environments that accelerate the initiation and progression of PDAC development [11], [19]–[22]. Overall, inflammation appears to impact the rate-limiting step in acinar cell transformation, which is the ability to assume a de-differentiated state [15]. How de-differentiation can turn an acinar cell into another pancreatic cell (such as a pancreatic β-cell), or a cell with cancerous potential (as in cases of deregulated Kras activity) is of great interest.

Despite inflammation being a hallmark component of exocrine injury, the impact of specific immune cells on acinar regeneration has remained elusive. Previous work has focused on early time points before de-differentiation has occurred [23]–[26], or does not address the *in vivo* role of immunological factors in a physiological system [27]. Our goal was to identify specific immune cells that impact pancreatic exocrine de-differentiation and regeneration in an *in vivo* injury model. We found the intensity of innate immune activation dictated the regenerative abilities of the pancreas by influencing the extent and maintenance of acinar de-differentiation. Development of non-resolving inflammation following caerulein-induced injury occurred with elevated pancreatic levels of neutrophils releasing interferon-γ (IFNγ) that increased the pro-inflammatory phenotype of F4/80^+^ macrophages. Ultimately, retention of this innate inflammation impaired pancreatic regeneration and enhanced de-differentiation of the pancreatic epithelium.

## Results

### Characterization of caerulein-induced injury and regeneration

**Figure 1:**
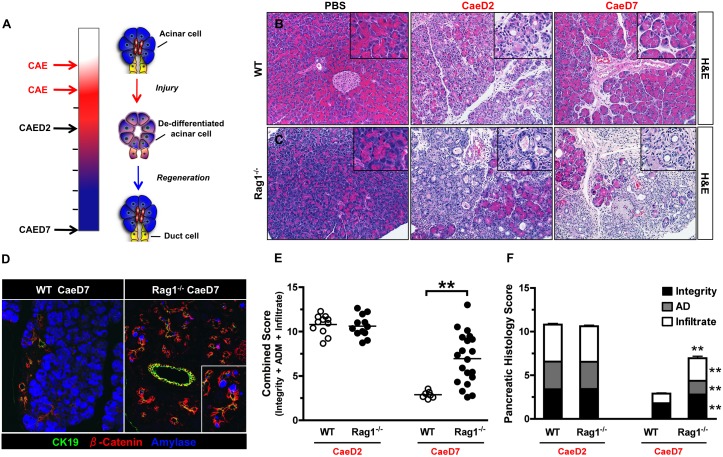
Pancreatic regeneration following acinar injury is impaired in Rag1^−/−^ mice. (A) Caerulein injection regime, time of tissue harvesting and schematic depiction of acinar de-differentiation and regeneration following caerulein-induced injury in WT mice. (B and C) Representative H&E staining of pancreas from WT and Rag1^−/−^ mice treated with PBS control or 2- and 7-days following caerulein-induced injury. (D) Pancreatic immuno-fluorescent staining for CK19, β-catenin and amylase from WT and Rag1^−/−^ mice at CaeD7. (E and F) Combined and individual scoring of pancreatic histological parameters from WT and Rag1^−/−^ mice at CaeD2 and CaeD7 (*n* = 5–20, *P<0.05, **P<0.01, *t* test).

Pancreatic injury is prompted by repeated administration of the cholecystokinin (CCK) analogue caerulein to stimulate acinar cells to overwhelmingly secrete digestive enzymes and transform into progenitor-like cells (Figure 1A) [9]–[11]. The induction of pancreatitis is initially characterized by an increase in the space between pancreatic lobes and cells, as well as an increase in the infiltration of immune cells. Pancreatic injury peaks 2-days after a 2-day injection regime (CaeD2) as acinar cells degranulate and morphologically adopt a duct-like architecture. We developed a pancreatic scoring system to evaluate and quantify the extent of changes in tissue integrity, acinar de-differentiation and immune infiltration (Figure S1, A–C). These morphological changes are accompanied by the re-expression of progenitor (Sox9, Hes1, FoxA2, Pdx1), stress (clusterin) and ductal factors (CK19) that allow de-differentiated acinar cells to adopt flexible properties (Figure S2A) [10]–[14]. In wild type (WT) mice, this de-differentiated state is transient, and the duct-like cells regenerate back into acinar cells within 7-days of injury (CaeD7) (Figure 1A, B and E, F) [10]–[14]. After CaeD7, the exocrine compartment of WT mice displays a histological and progenitor expression profile similar to that of PBS control mice.

### Regeneration is impaired in the absence of B and T cells

To address whether adaptive immune cells are required for acinar de-differentiation and/or subsequent regeneration, we treated Rag1^−/−^ mice (devoid of mature B and T cells) with caerulein [28]. WT and Rag1^−/−^ mice both undergo acinar de-differentiation at CaeD2 at similar levels, suggesting B and T cells are dispensable for this process (Figure 1B, C and E, F). However, an impairment in regeneration was seen in the absence of adaptive immune cells as suggested by areas of a de-differentiated ductal epithelium (visualized by CK19 and β-catenin staining) residually expressing amylase that replace the normal exocrine compartment in Rag1^−/−^ mice at CaeD7 (Figures 1D and Figure S2B). Fourteen-days after caerulein treatment (CaeD14), the pancreatic epithelium continues to become more duct-like and markers associated with pancreatic progenitor cells including, Ngn3, insulin and Nkx2.2, can be detected (Figure S3A–B). Expression of markers that are normally absent in mature exocrine cells, as well as in the transient ductal intermediates seen at CaeD2, suggests enhanced de-differentiation of the ductal epithelium and the potential to give rise to cells of other pancreatic lineages [29], [30]. Ultimately, these de-differentiated areas are replaced by adipocytes by CaeD24, supporting an overall defect in tissue regeneration (Figure S3C).

While the majority of the pancreas is affected in both genotypes at CaeD2, the pancreas of Rag1^−/−^ mice at CaeD7 is composed of variable regions of either normal or damaged tissue (Figure 1B, C and E, F). This lack of uniformity suggests lack of adaptive immune cells renders Rag1^−/−^ mice susceptible to developing isolated inflammatory pockets that are unable to regenerate normally. Knockout mice and adoptive transfer studies using B and T subsets indicate this was not exclusively a B or T cell dependent process since either subset alone led to regeneration similar to that of WT mice (data not shown). Moreover the ability of some Rag1^−/−^ mice to regenerate at CaeD7 (Figure 1E, F) suggested impaired regeneration in Rag1^−/−^ mice may be a result of aberrant activation of the innate immune system.

### Macrophages promote and maintain acinar de-differentiation

**Figure 2:**
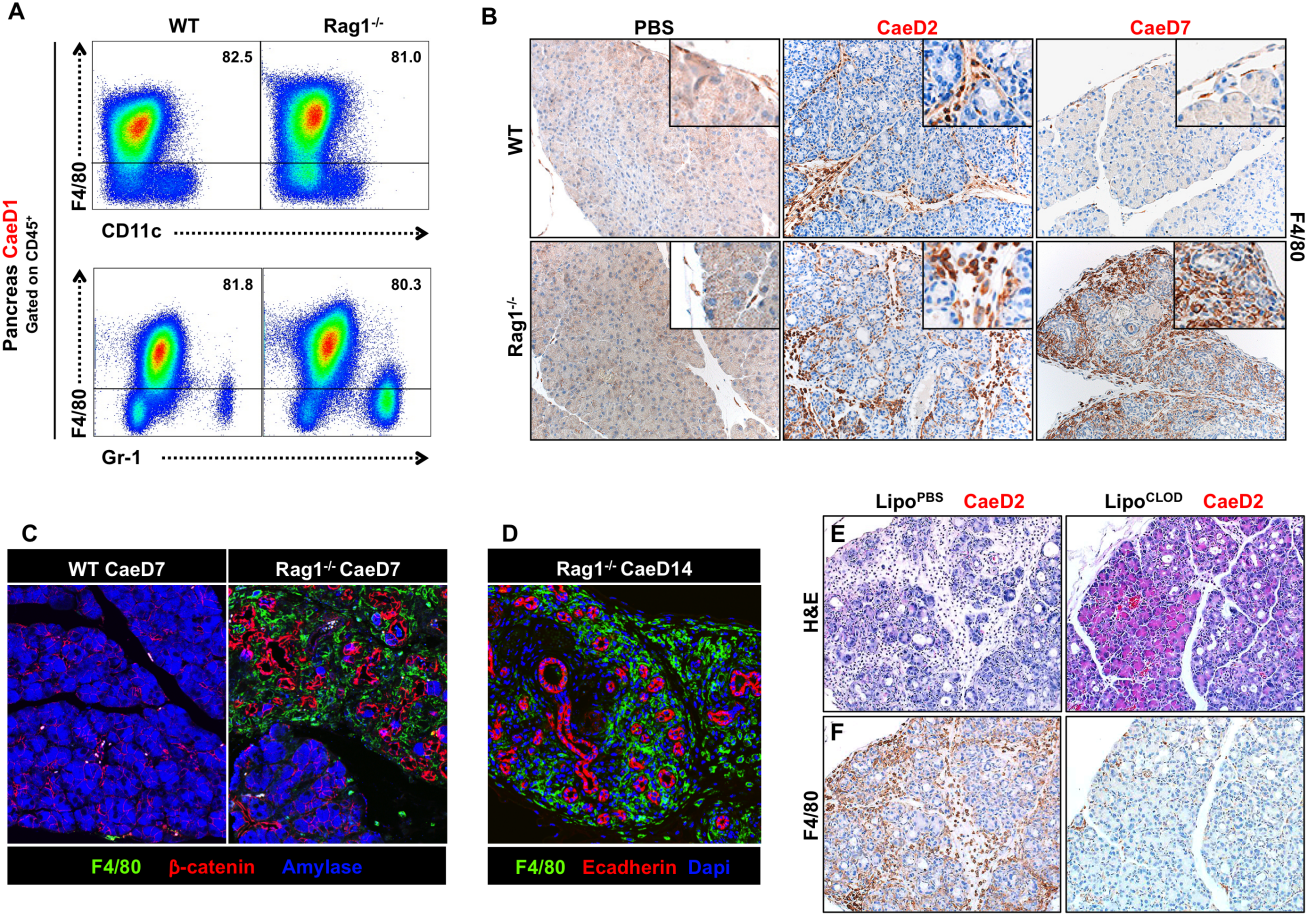
Macrophages are the predominant immune cell infiltrating the pancreas following caerulein-induced injury. (A) Representative FACS plot showing the percentage of CD45^+^ cells that are F4/80^+^ compared to F4/80^−^CD11c^+^ dendritic cells and F4/80^−^Gr1^+^ myeloid cells in the pancreatic immune infiltrate at CaeD1. (B) Immuno-histochemical staining of F4/80^+^ cells from WT and Rag1^−/−^ treated with PBS or caerulein. (C and D) Immuno-fluorescent staining of the pancreas from WT and Rag1^−/−^ mice showing F4/80^+^ cells are associated with areas of the de-differentiated epithelium (visualized by β-catenin and Ecadherin) present only in Rag1^−/−^ mice 7-days after caerulein treatment and completely surrounding the lobular epithelium within 14-days. (E and F) Representative H&E and corresponding areas stained with F4/80 from the pancreas of Rag1^−/−^ mice treated with liposomes filled with either PBS or clodronate during caerulein treatment and harvested 2-days after the last caerulein injection.

Macrophages (F4/80^+^) are components of the innate immune system that phagocytose dead/damaged cells and contribute to tissue remodeling and regeneration following injury [4]. We found F4/80^+^ macrophages were the predominant immune cells present in the pancreas of both WT and Rag1^−/−^ mice at CaeD1 (Figure 2A), while continued macrophage infiltration was detected only in Rag1^−/−^ mice at CaeD7 (Figure 2B). The majority of macrophages were located adjacent to de-differentiated acinar cells (Figure 2C), with entire lobes of the normal exocrine compartment eventually being replaced by a de-differentiated ductal epithelium surrounded by macrophages (Figures 2D and S3A–B). Depletion of macrophages in Rag1^−/−^ mice using clodronated liposomes [31] decreased the extent of acinar de-differentiation, with areas not associated with macrophages consistently displaying less de-differentiation (Figures 2E–F and S4A–C). These data support the involvement of F4/80^+^ macrophages in promoting and maintaining acinar de-differentiation.

### Rag1^−/−^ mice have increased pro-inflammatory macrophages

**Figure 3:**
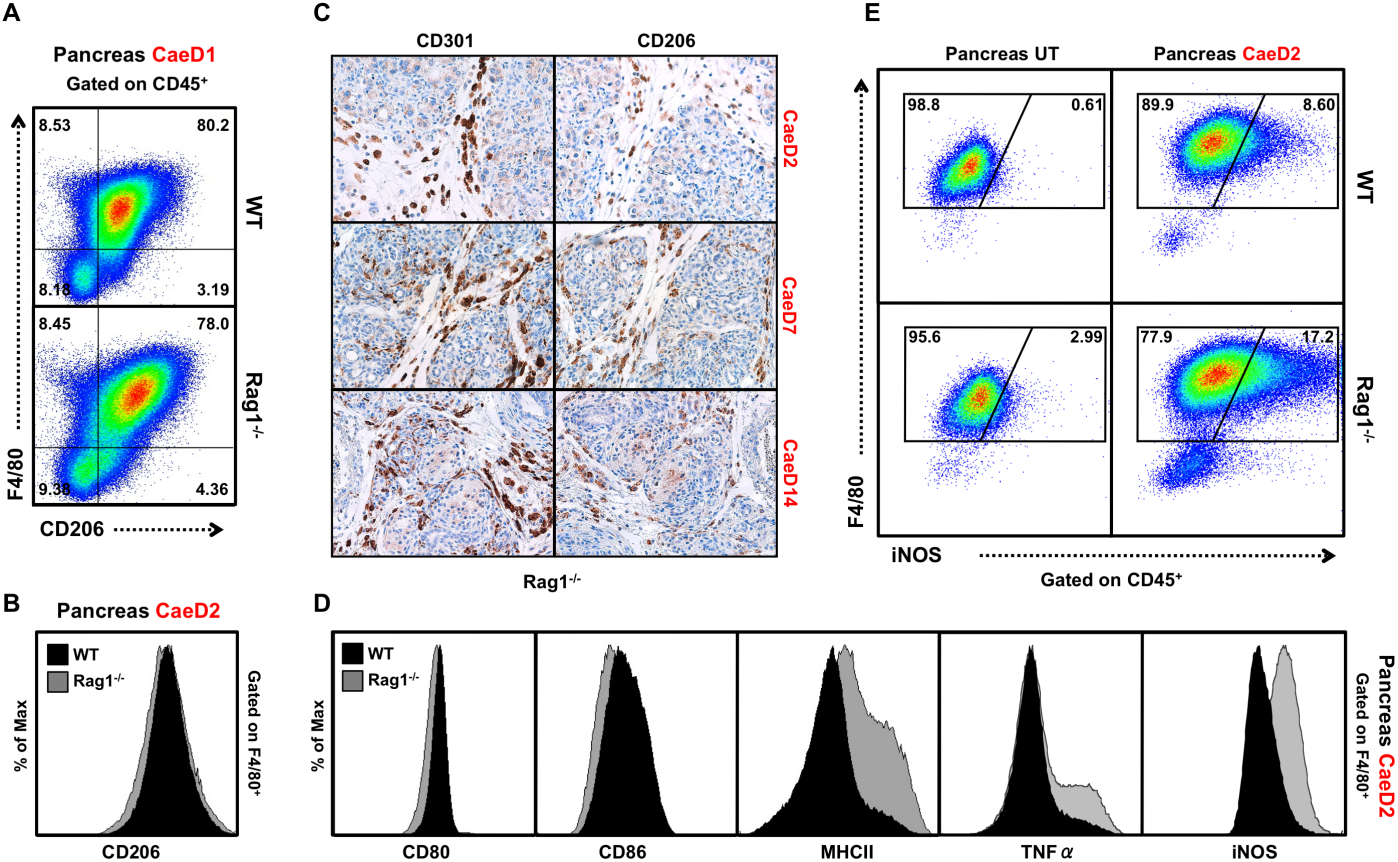
Inflammatory activation of macrophages is increased in Rag1^−/−^ mice before and after injury. (A and B) Representative FACS plots characterizing expression of the CD45^+^ immune cells that are F4/80^+^ and CD206^+^ in the pancreatic immune infiltrate at CaeD1 and CaeD2 (*n* = 8). (C) Representative immuno-histochemical staining of CD301^+^ and CD206^+^ cells in Rag1^−/−^ mice treated with caerulein at designated time points. (D) Representative FACS plots characterizing F4/80^+^ macrophages isolated from the pancreas of WT and Rag1^−/−^ mice at CaeD2 (*n* = 4–8). (E) Representative FACS plots of iNOS expression from macrophages isolated from the pancreas of WT and Rag1^−/−^ mice in untreated and CaeD2 treated mice (*n* = 4–8).

Macrophages are known to express either inflammatory (M1, classical activation), or anti-inflammatory (M2, alternatively activated) [3], [4], [32], [33] characteristics in response to their environment. Macrophages associated with tissue injury display elevated levels of M1 markers such as pro-inflammatory cytokines, iNOS, CD86, and MHC class II (MHCII) [33], [34], while alternatively activated M2 macrophages have increased expression of the mannose receptor (CD206) and are involved in promoting tissue repair [33], [34]. Infiltrating macrophages were measured for inflammatory (TNFα, iNOS, CD80, CD86, MHCII expression) and anti-inflammatory (CD206, CD301) markers to determine whether Rag1^−/−^ mice had changes in the proportion of M1 inflammatory versus M2 anti-inflammatory properties following injury. Pancreatic macrophages from both genotypes expressed similar levels of the M2 marker CD206 (Figure 3A–B) by FACS analysis, and this was confirmed by the comparable immuno-histochemical staining of M2 markers CD206 and CD301 at CaeD2-, 7- and 14-days (Figure 3C). However, an increase in the M1 markers MHCII, iNOS and TNFα was detected in macrophages from Rag1^−/−^ mice, with no difference observed in CD80 and CD86 at CaeD2 (Figure 3D). The elevated levels of intracellular iNOS and TNFα (Figure 3D–E) in pancreatic macrophages from Rag1^−/−^ mice indicate an M1 shift in the macrophage balance that creates a more inflammatory pancreatic environment after injury.

### Rag1^−/−^ mice have increased pancreatic levels of γPMNs

**Figure 4:**
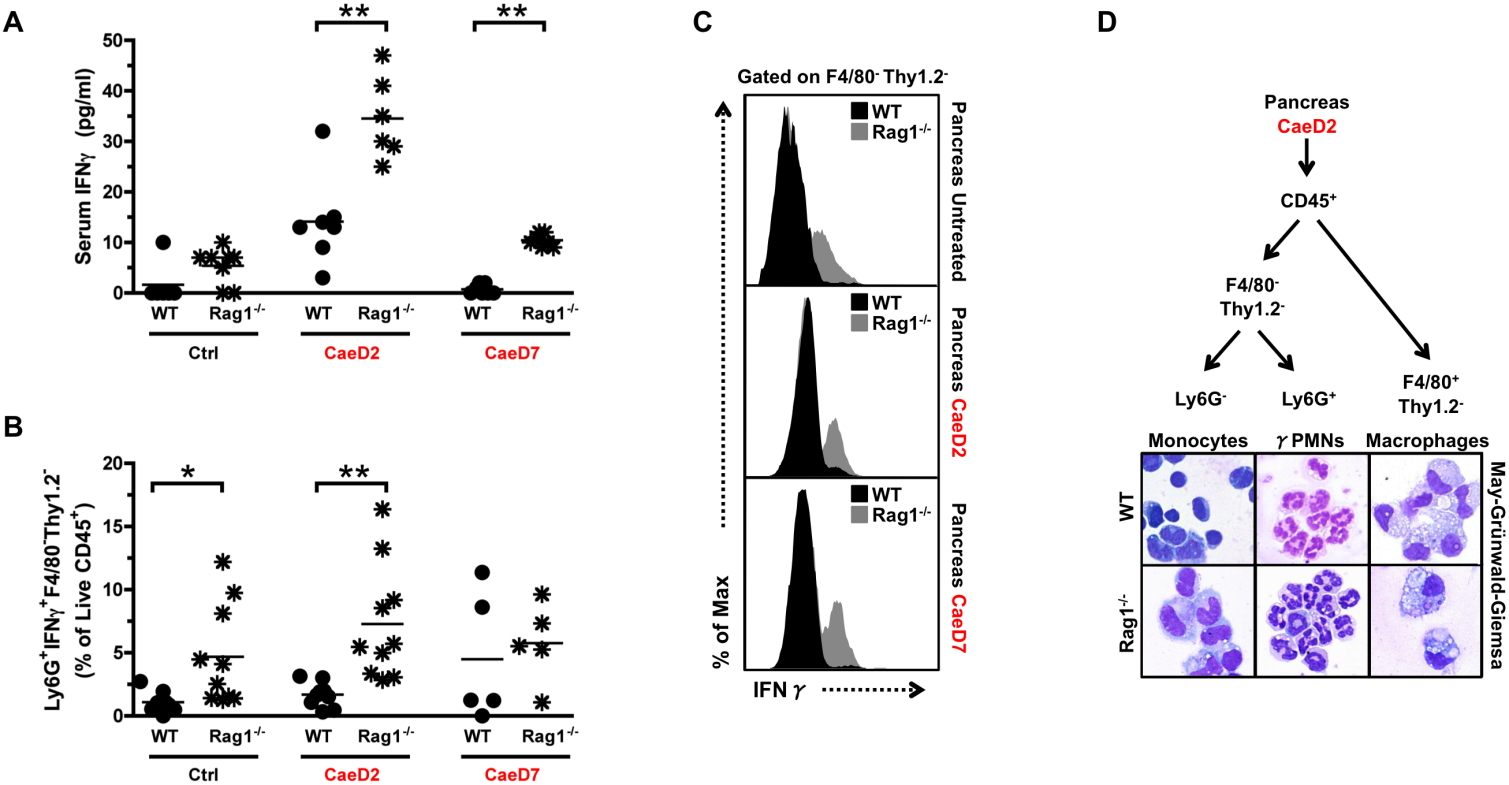
γPMNs are present during caerulein-induced injury in WT mice and are elevated in Rag1^−/−^ mice. (A) IFNγ levels in serum isolated from WT and Rag1^−/−^ mice with and without caerulein treatment (*n* = 6–8, *P<0.05, **P≤0.01, *t* test). (B) FACS analysis of the percentage of F4/80^−^Thy1.2^−^ cells expressing Ly6G^+^ and IFNγ^+^ in the pancreas of WT and Rag1^−/−^ mice with or without caerulein treatment (*n* = 5–8, *P<0.05, **P<0.01, *t* test). (C) Percentage of Ly6G^+^IFNγ^+^ (CD45^+^F4/80^−^Thy1.2^−^) amongst live CD45^+^ cells infiltrating the pancreas with or without caerulein treatment as indicated. (D) Sort strategy isolating indicated populations from the pancreas of WT and Rag1^−/−^ mice and cytospin preparation visualizing May-Grünwald-Giemsa staining.

IFNγ is a potent inflammatory cytokine known to activate M1 type macrophages and promote an inflammatory response [24]–[26]. Additionally, the development of a similar ductal epithelium with progenitor properties in a mouse model over-expressing IFNγ in pancreatic β-cells [35] prompted us to investigate whether elevated levels of IFNγ in Rag1^−/−^ mice were contributing to the formation of duct-like cells following caerulein-induced injury. Interestingly, an increase in serum levels of IFNγ in both genotypes was detected after injury, with noticeably higher levels of IFNγ seen before and after caerulein treatment in Rag1^−/−^ mice (Figure 4A). Furthermore, Rag1^−/−^ mice have a higher percentage of Ly6G^+^ cells expressing IFNγ that are not macrophages (F4/80^−^), nor innate lymphoid cells (Thy1.2^−^) within the pancreas (Figures 4B–C and S5A). Further characterization determined the F4/80^−^Ly6G^+^ cells express CD11b (an integrin family member involved in leukocyte adhesion and migration), as well as low levels of the chemokine receptor CCR2 (Figure S5B), but lack the expression of the natural killer (NK) cell marker CD49b (Figure S5C). Lastly, ability to secrete IFNγ was confirmed by measuring the amount of IFNγ in the media from cultured F4/80^−^Ly6G^+^ fractionated cells (Figure S5D). The detection of Ly6G^+^IFNγ^+^ cells in the pancreas of WT mice at CaeD2 and CaeD7 indicates these cells are part of the normal response to caerulein-induced injury (Figure 4B–C), while the elevated levels in the pancreas of untreated, CaeD2 and CaeD7 Rag1^−/−^ mice suggested these cells may contribute to the prolonged innate inflammatory response seen in Rag1^−/−^ mice. In contrast to the monocytic cells present in the F4/80^−^Thy1.2^−^Ly6G^−^ fraction (containing some NK and B cells) (Figure 4D), FACS sorted Ly6G^+^ cells have a poly-morphonuclear (PMN) cytoplasm, leading us to refer to these IFNγ^+^Ly6G^+^CCR2^+^F4/80^−^Thy1.2^−^ cells as γPMNs (Figure 4D). PMNs, also known as neutrophils, are innate immune cells that are typically the first leukocyte recruited to inflammatory sites [36]. PMNs are recognized as major effectors of acute inflammation, and the elevated levels of γPMNs in Rag1^−/−^ mice further supported involvement of these cells in influencing the immunological response of macrophages [25], [37].

### IFNγ-induced macrophage expression of iNOS enhances inflammation

**Figure 5:**
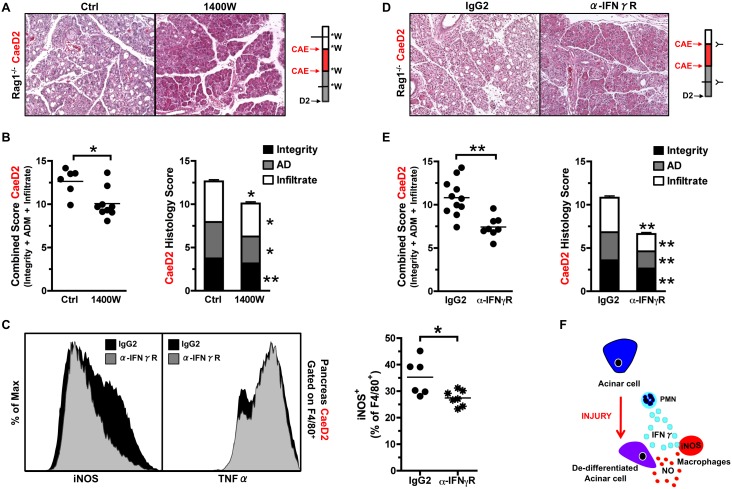
Decreasing inflammatory macrophage activation improves pancreatic injury. (A) H&E staining of pancreas from Rag1^−/−^ mice treated with control or the iNOS inhibitor 1400 W 2-days following caerulein-induced injury. Injection regime of 1400 W (W*) is indicated in adjacent cartoon. (B) Combined and individual pancreatic histology scores from Rag1^−/−^ mice treated with control or iNOS inhibitor 1400 W (*n* = 6–9). (C) Representative FACS analysis of macrophages expressing iNOS isolated from the pancreas of Rag1^−/−^ mice treated with α-IFNγR and IgG2 isotype control at CaeD2 (*n* = 4–8). (D) H&E staining of pancreas from Rag1^−/−^ mice treated with α-IFNγR and IgG2 isotype control at CaeD2. Injection regime of α-IFNγR (Y) is indicated in adjacent cartoon. (E) Combined and individual pancreatic histology scores from Rag1^−/−^ mice treated with α-IFNγR and IgG2 isotype control (*n* = 8–11). (F) Schematic depicting how increased innate inflammation impacts exocrine de-diferentiation.

Macrophage-mediated tissue injury results from the synthesis and release of molecules with pro-inflammatory and cytotoxic activity. Reactive oxygen species (ROS) are generated via enzyme-catalyzed reactions during mitochondrial respiration that, under physiological conditions, are important for maintaining tissue homeostasis. However, if ROS are produced in large quantities or without control, oxidative stress can enhance the extent of tissue injury [34]. ROS production in macrophages occurs via the enzyme iNOS, suggesting inhibition of iNOS may mitigate caerulein-induced injury [34]. Upon administration of the iNOS inhibitor 1400 W, the degree of tissue integrity, acinar de-differentiation and immune infiltration were decreased significantly, improving the overall extent of pancreatic injury in Rag1^−/−^ mice at CaeD2 (Figure 5A–B). Furthermore, IFNγ-blockade in Rag1^−/−^ mice using an antibody directed against the IFNγR decreased macrophage expression of iNOS (Figure 5C), as well as significantly improved all aspects of pancreatic injury at CaeD2 compared to IgG2 isotype control (Figure 5D–E). Collectively these data indicate that IFNγ release from PMNs promotes an increased inflammatory activation in macrophages, leading to the de-differentiation of the pancreatic epithelium (Figure 5F).

## Discussion

**Figure 6:**
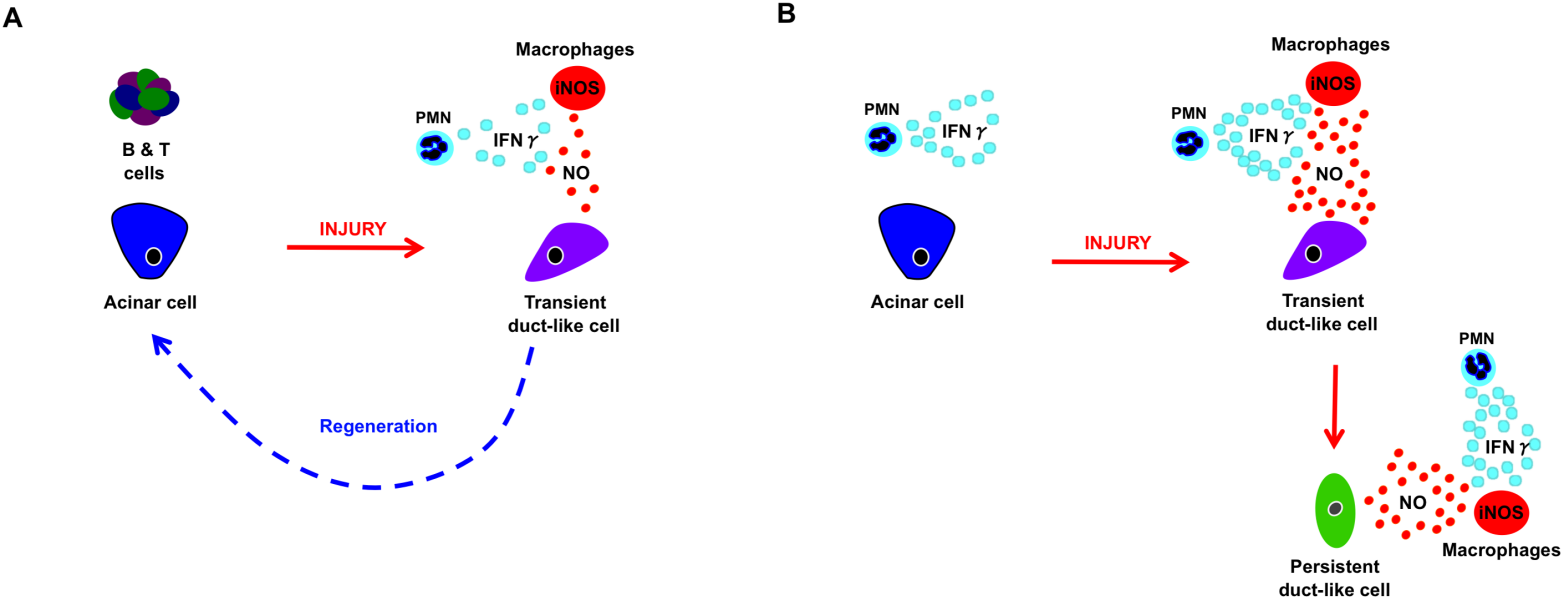
Inflammatory factors that influence effective versus ineffective pancreatic regeneration. Schematic depicting the impact increased innate inflammation has on exocrine de-differentiation and regeneration in (A) the presence (WT mice) and absence (B) of adaptive immune cells (Rag1^−/−^ mice).

Mature acinar cells acquire plastic characteristics and lose restrictions that limit cellular identity in response to injury. This instability creates an opportunity where a cell can either be directed into a desired fate of choice, or undergo unregulated cancerous neoplasia in the presence of oncogene expression. Understanding factors that manipulate this process can improve therapeutic interventions aimed at generating new tissue, as well as preventing unwanted cellular transformation. Here we determine distinct immune populations that influence the regenerative abilities of the pancreas following injury. We find that innate immune macrophages and γPMNs promote and maintain acinar de-differentiation, while adaptive immune cells support normal regeneration (Figure 6A). In the absence of B and T cells, pancreatic levels of γPMNs and systemic levels of IFNγ are elevated before and in response to caerulein-induced pancreatic injury. Macrophages recruited to the pancreas possess both M1 and M2 characteristics, with Rag1^−/−^ mice displaying elevated levels of iNOS and other pro-inflammatory factors. As a result, over-ignition of the innate inflammatory response leads to prolonged and isolated inflammatory pockets that are unable to regenerate and differ greatly from areas of normal tissue. Macrophage retention enhances acinar de-differentiation, causing the exocrine compartment to be replaced by a ductal epithelium containing progenitor-like cells with possible multi-potent potential (Figure 6B). Recent studies support the idea that exocrine injury and/or inflammation can lead to β-cell neogenesis in the adult pancreas [38], [39], and the inflammation-induced formation of a de-differentiated pancreatic epithelium with progenitor characteristics supports this idea. However, these areas appear to be destroyed in Rag1^−/−^ mice with persistent inflammation, making it unclear whether these cells can actually give rise to an endocrine lineage.

The chronic inflammation seen in Rag1^−/−^ mice following injury indicates adaptive immune cells are required for normal tissue regeneration and dissipation of innate immune infiltration. Previous studies have supported a role for adaptive immune cells in the direct suppression of innate immune responses following inflammation and injury [40]–[43]. In our study, genetic knockout mice, adoptive transfer studies and the variability in regeneration seen in Rag1^−/−^ mice following caerulein-induced injury indicate these mice are able to regenerate, but are more susceptible to over-activation of the innate inflammatory response. While adaptive immune cells most likely regulate the innate immune responses to injury in many ways, it does appear B and T cells may exert a systemic regulation over innate immunity by stabilizing the inflammatory environment and limiting numbers of innate populations such as γPMNs (Figure 6). Notably, the patchy areas of injury scattered across the pancreas highlights how the inflammatory milieu can influence the differentiation state of pancreatic epithelial cells and create distinct areas that differ from the rest of the tissue. These micro-environments may provide conflicting signals to immune cells (macrophages and PMNs in particular) that contribute to the formation of unfavorable immune responses in disease states such as cancer and auto-immunity.

Ultimately, chronic inflammation is known to contribute significantly to the pathogenesis of several diseases including atherosclerosis, obesity, cancer and neurodegenerative diseases [4], [7], [8]. The problem does not appear to be the initiation of inflammation, but rather the inability to resolve the response after it has begun. In several cases, propagation of inflammation triggered by tissue injury is thought to develop into non-resolving inflammation [4], [7], [8]. Innate cells are usually the first immune cells to respond to tissue injury, and aberrant activity of these populations has been implicated in several disease states associated with chronic inflammation [4], [7], [8], [44]. Being able to target select aspects of chronic inflammation, but avoiding others, is the real challenge to effective therapeutic design of anti-inflammatory therapies. This work highlights how an integrated therapeutic strategy that accounts for the interaction between the immune cells and the tissue microenvironment may be an effective approach to interrupt the propagation of inflammation in localized areas.

## Experimental Procedures

### Mice

WT and Rag1^−/−^ on a C57.Bl6 background were purchased from Jackson Lab. All mouse experiments complied with the Animal Welfare Act and the National Institutes of Health guidelines for the ethical care and use of animals in biomedical research and were performed under the approval of the University of California at San Francisco Institutional Care and Use of Animals Committee (IACUC).

### Caerulein treatment

Non-necrotic acute pancreatitis was induced in 6–7 week old mice (ranging 20–25 grams) as previously described [10], [11]. Briefly, every hour 1 intraperitoneal injection of caerulein or PBS was administered for 8 hours (2 µg/injection) for 2 consecutive days. The final day of caerulein injection was considered day 0.

### Pancreatic Histological Quantification

Pancreatic damage was quantified by scoring 9 randomly chosen images from 3 slides spanning 300–600 µm for a total of 27 images/mouse. Tissue integrity, acinar de-differentiation and infiltration were scored from (0–5) and individual scores for these three categories were added to make a combined score. Definition and examples of parameters measured are depicted in Figure S1A–C and Figure S4C.

### Immunohistochemistry and immunofluorescence

Pancreata were fixed for 12–16 hours in Z-Fix (Anatech Ltd.), embedded in paraffin, cut into 5-µm–thick sections, and placed on Superfrost Plus slides (Fisher Scientific). Sections were subjected to H&E, immunohistochemical, and immunofluorescent staining as previously described [11]. The following primary antibodies were used: rabbit α-amylase (1∶200; Sigma-Aldrich), rat α-CK19 (TROMAIII, 1∶200 dilution), guinea pig α-Pdx1 (1∶200; gift from Michael German, UCSF), goat α-clusterin (1∶200; Santa Cruz Biotechnology Inc.), rabbit α-Sox9 (1∶200; Chemicon), mouse α– β-catenin (1∶200; BD), rat α-CD206 (1∶200; Serotec), rat α-CD301 (1∶200; Serotec), rat α-F4/80 (1∶200; Abcam), mouse α-e-cadherin (1∶200; BD), rabbit α-Ngn3 (1∶200; gift from Michael German, UCSF), mouse α-Nkx2.2 (1∶200; Hybridoma Bank, University of Iowa) and Guinea pig α-insulin (1∶500; Linco). Biotinylated α-rabbit (Vector Labs), α-goat and α-rat (Jackson ImmunoResearch Laboratories Inc.) antibodies were used as secondary antibodies at a 1∶200 dilution for immunohistochemistry using 3-3′-Diaminobenzidine tetrahydrochloride (Vector Labs) as a chromogen. Alexa Fluor 488, 633 and 555 secondary antibodies (Molecular Probes; Invitrogen) were used at a 1∶200 dilution. Confocal images were collected on a Leica SP2 microscope at consistent gain and offset settings, while bright-field images were acquired using a Zeiss Axio Imager D1 scope.

### FACS antibodies and Reagents

FACS experiments used the following antibodies for staining: CD45 (30-F11), CD11b (M1/70), CD11c (N418), CD206 (MR5D3), F4/80 (BM8), Ly6G (1A8), CCR2 (475301), Thy1.2 (53-2.1), CD49b (DX5), NK1.1 (PK136), IFNγ (XMG1.2), iNOS (C-11), TNFα (MP6-XT22) and specific isotype matched control (BioLegend, BD PharMingen, Invitrogen, eBioscience, LifeSpan Biosciences, Santa Cruz Biotechnology or the University of California at San Francisco Monoclonal Antibody Core). Live/Dead fixable stain (Invitrogen) and DAPI (Sigma) were used to determine live cells.

### Flow cytometry and cell sorting

Single cell suspension of mouse pancreas and peritoneal cavity (PerC) were prepared as previously described [45], [46]. Cells were fractioned by centrifugation through a 40%–60% (vol/vol) percoll gradient to enrich for innate (upper layer) and adaptive (lower layer) immune cells. Cells were blocked with αCD16/32 (University of California at San Francisco Monoclonal Antibody Core) and rat serum in FACS buffer (2% FBS, 2 mM EDTA in PBS) before surface staining (20–60 min) and ran on a LSRII instrument and FACSDiva software (BD Biosciences), and analyzed using Flowjo software (Treestar). Cells were all sorted on a MoFlo cytometer (Beckman Coulter) to >95% purity.

### Cytokine production

The cell fractions from percoll enrichment were individually incubated in non-tissue culture treated 24 well plates (BD) in DMEM medium (5% FBS+pen/strp) supplemented with 1 µl/ml Golgi Plug (BD Biosciences). After 3-hours at 37°C, 5%CO_2_, cells were pelleted, washed and blocked with αCD16/32 (UCSF hybridoma Core) before staining. Cells were treated with Cytofix/Cytoperm (BD Biosciences) prior to intracellular staining.

### Cytopsin

The lower layer from percoll enrichment was stained as indicated in Figure 4D and used for isolation of γPMNs, while the upper layer was used for macrophage isolation. Cells were sorted on a MoFlo cytometer and given to the UCSF Helen Diller Family Comprehensive Cancer Center Mouse Pathology core for cytospin and staining.

### Liposome Preparation

Multilamellar liposomes were prepared as previously described [31]. Briefly, 86 mg of phosphatidylcholine (egg lecitin; a gift from Lipoid) and 8 mg of cholesterol (Sigma, St. Louis, MO) were dissolved in 10 ml chloroform in a 500-ml round-bottom flask and dried by low-vacuum rotary evaporation (Büchi, Rotavapor). Liposomes were encapsulated with clodronate (Sigma) or PBS. For macrophage depletion, Rag1^−/−^ mice received both an i.v. and an i.p. injection of either PBS or clodronated loaded liposomes (∼250 µl containing 2.35 mg lipid) 2 times a day (8 hours apart) for 3 days. The first injection began 24 hours before initiation of caerulein injections.

### *In vivo* treatments

The iNOS inhibitor 1400 W (Cayman Chemicals) was prepared fresh daily in PBS and 10 mg/kg body weight was injected 1–2 times daily for 3 consecutive days starting 1 day before caerulein treatment (Figure 5A). The following antibodies were used for antibody depletion experiments: α-IFNγR (GR-20), Rat IgG2a (2A3) (University of California at San Francisco Monoclonal Antibody Core; Bio-X-Cell). Mice received i.p. injections of 0.5 mg of α- IFNγR (GR-20) and Rat IgG2a (2A3) isotype control every other day (Figure 5D).

### Elisa

IFNγ was performed as suggested by the manufacturer (eBioscience). Splenocytes were isolated from WT mice and incubated at 37°C in non-tissue culture treated 24 well plates (BD) in DMEM medium (5% FBS+pen/strp) supplemented with 0.5 M ionomycin, 10 ng/ml of PMA. The lower layer from percoll enrichments from WT CaeD2 treated mice were incubated in non-tissue culture treated 24 well plates (BD) in DMEM medium (5% FBS+pen/strp). After 3–4 h, cells were pelleted and media was collected for Elisa assay.

## Supporting Information

Figure S1 **Pancreatic histological scoring system.** (A–C) Three parameters were evaluated: lobular integrity, acinar de-differentiation (AD) and immune infiltration. Scoring is based on an incremental scale, increasing with severity and images exemplifying the defined criteria as shown.
(TIF)

Figure S2 **Pancreatic epithelial expression of progenitor markers is prolonged in Rag1^−/−^ mice following injury.** (A and B) Expression of progenitor/stress markers Sox9 and Clusterin are detected in both WT and Rag1^−/−^ mice at CaeD2 but only in Rag1^−/−^ mice at CaeD7.
(TIF)

Figure S3 **De-differentiation of the pancreatic epithelium is enhanced in Rag1^−/−^ mice.** (A) Co-expression of insulin and Nkx2.2 and (B) expression of Pdx1 and Ngn3 can be detected in cells associated with the CK19^+^ ductal epithelium of Rag1^−/−^ mice at CaeD14. Arrows indicate areas magnified in inset. (C) H&E staining of pancreatic epithelium in Rag1^−/−^ mice at CaeD14 and CaeD24 showing morphological characteristics of adipocytes.
(TIF)

Figure S4 **Macrophages depletion improves extent of caerulein-induced injury.** (A) Percentage of F4/80^+^ macrophages present among CD45^+^ immune cells in the peritoneal cavity (PerC) and spleen from Rag1^−/−^ mice treated with liposomes containing PBS or clodronate at CaeD2; *n* = 3 mice per group. (B) Pancreatic histology score evaluating the degree of acinar de-differentiation and F4/80^+^ infiltration (*n* = 3, *P<0.05, **P≤0.01, *t* test). (C) Representative images demonstrating scoring system used to evaluate the extent of F4/80^+^ infiltration.
(TIF)

Figure S5 **Phenotype of γPMNs.** (A–C) Representative FACS analysis showing marker expression characterizing Ly6G^+^IFNγ^+^ cells. (A) Ly6G^+^IFNγ^+^ cells and isotype control (A), IFNγ^+^ cells are CCR2^+^ and CD11b^+^ (B), but not CD49b^+^ (C). (D) IFNγ levels present in media from cultured spleen cells +/− stimulation to release IFNγ and density fraction containing γPMNs isolated from the pancreas of WT mice at CaeD2 (*n* = 4).
(TIF)
