# Supplementary figures and images for: Aberrant Innate Immune Activation following Tissue Injury Impairs Pancreatic Regeneration
Source: PLoS One. 2014 Jul 10;9(7):e102125. doi: 10.1371/journal.pone.0102125 (PMC4092101; doi:10.1371/journal.pone.0102125)

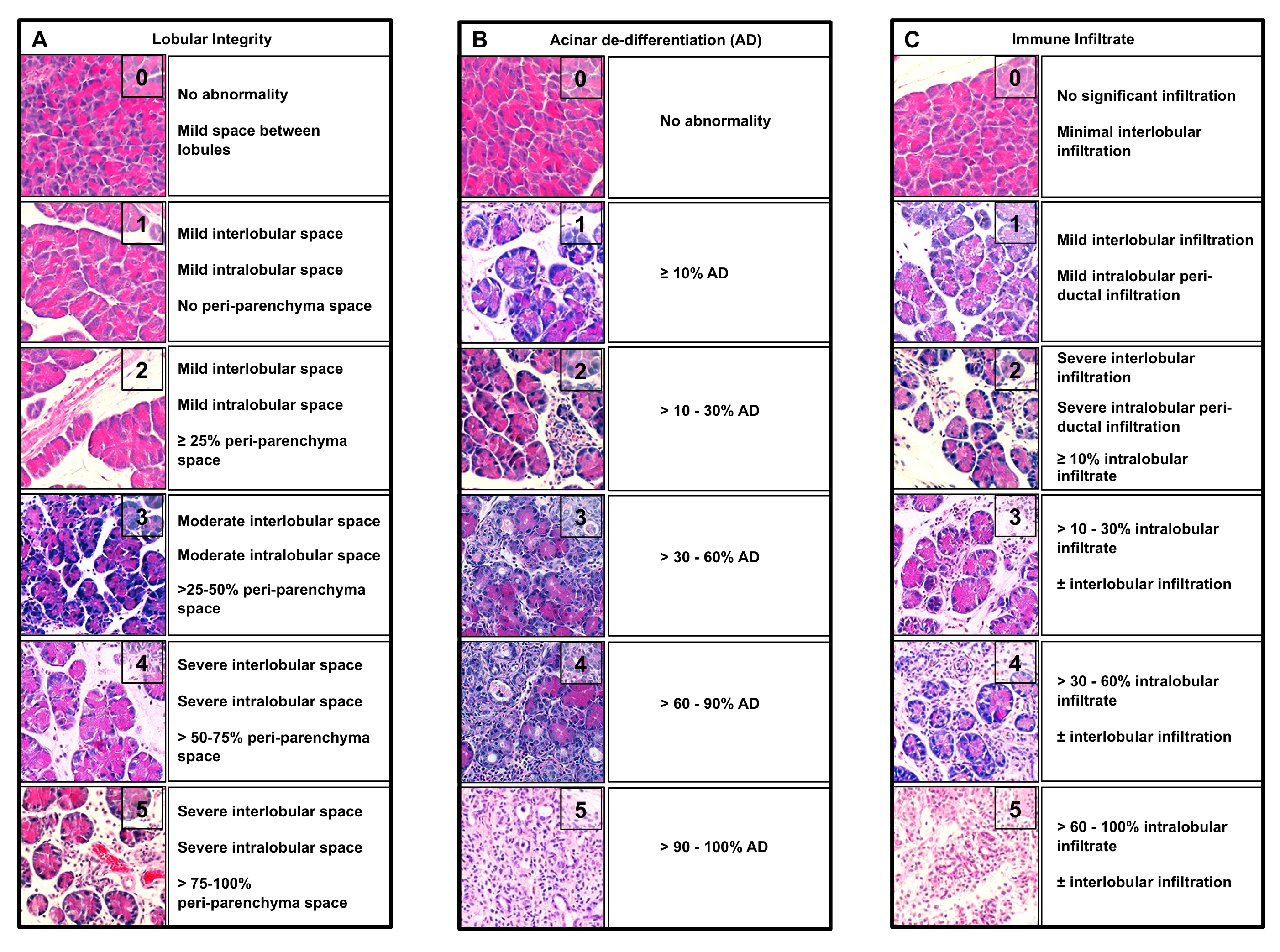

Supplement: Figure S1 — Pancreatic histological scoring system. (A–C) Three parameters were evaluated: lobular integrity, acinar de-differentiation (AD) and immune infiltration. Scoring is based on an incremental scale, increasing with severity and images exemplifying the defined criteria as shown. (TIF) [file pone.0102125.s001.tif]

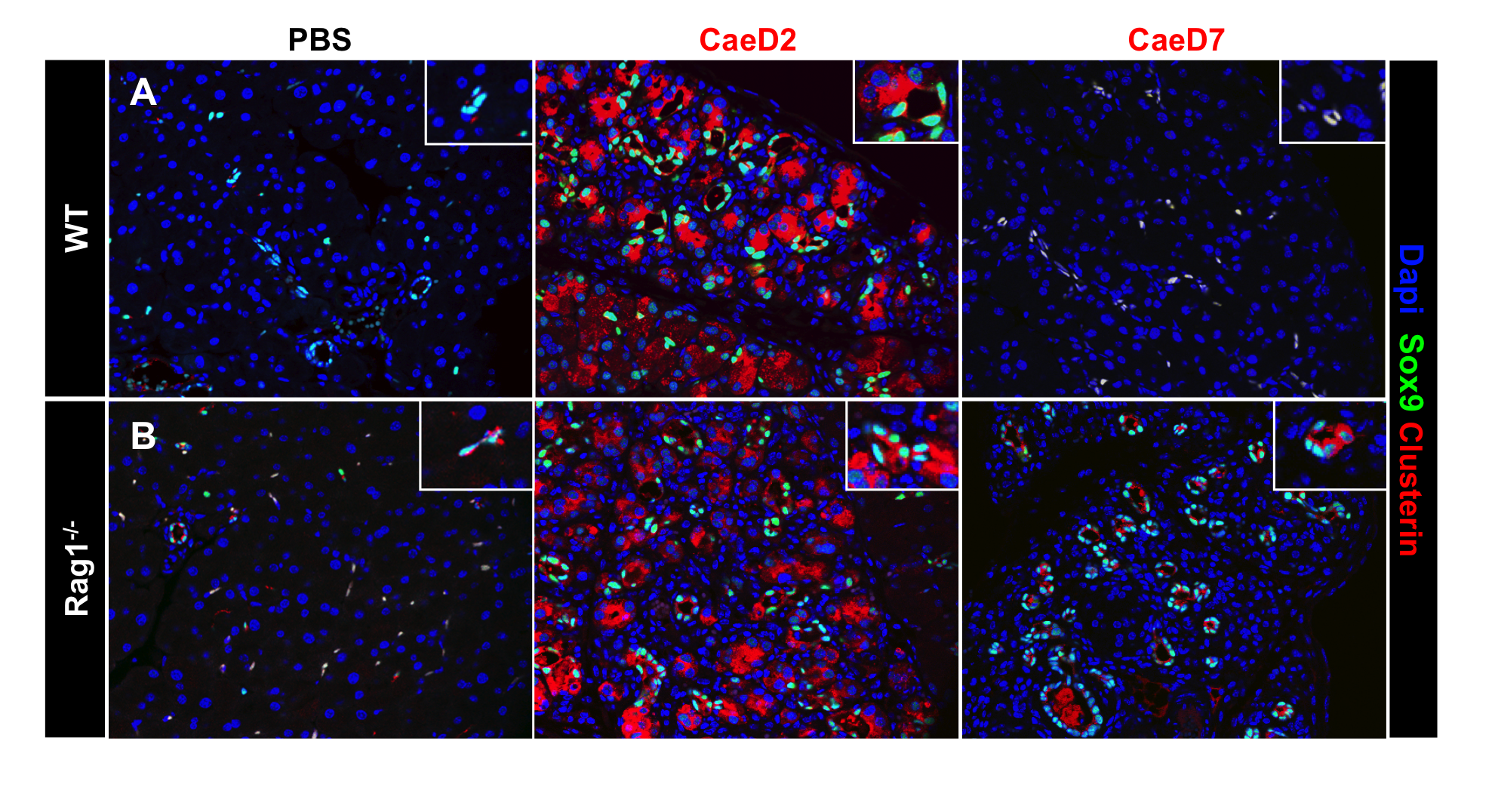

Supplement: Figure S2 — Pancreatic epithelial expression of progenitor markers is prolonged in Rag1−/− mice following injury. (A and B) Expression of progenitor/stress markers Sox9 and Clusterin are detected in both WT and Rag1−/− mice at CaeD2 but only in Rag1−/− mice at CaeD7. (TIF) [file pone.0102125.s002.tif]

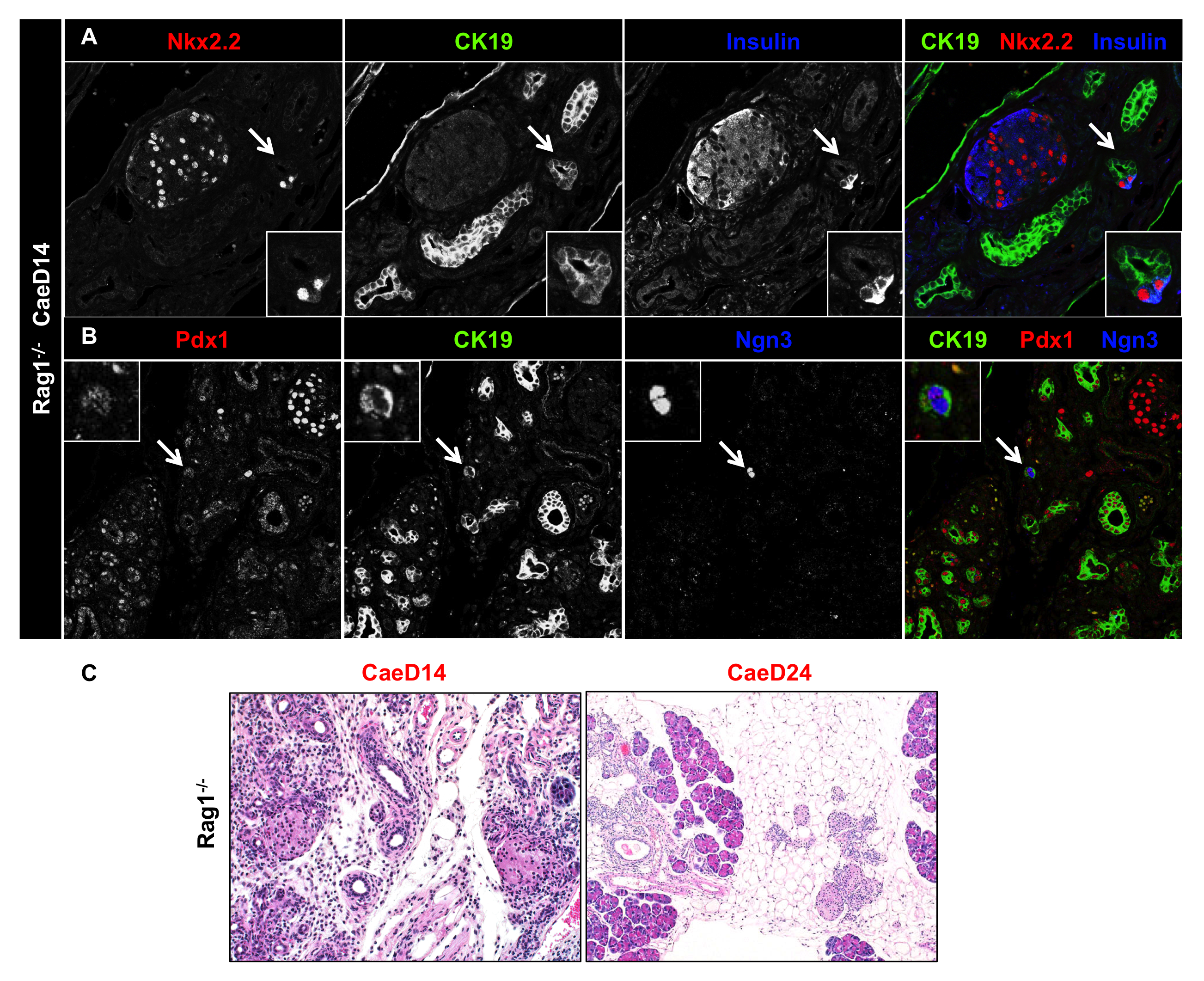

Supplement: Figure S3 — De-differentiation of the pancreatic epithelium is enhanced in Rag1−/− mice. (A) Co-expression of insulin and Nkx2.2 and (B) expression of Pdx1 and Ngn3 can be detected in cells associated with the CK19+ ductal epithelium of Rag1−/− mice at CaeD14. Arrows indicate areas magnified in inset. (C) H&E staining of pancreatic epithelium in Rag1−/− mice at CaeD14 and CaeD24 showing morphological characteristics of adipocytes. (TIF) [file pone.0102125.s003.tif]

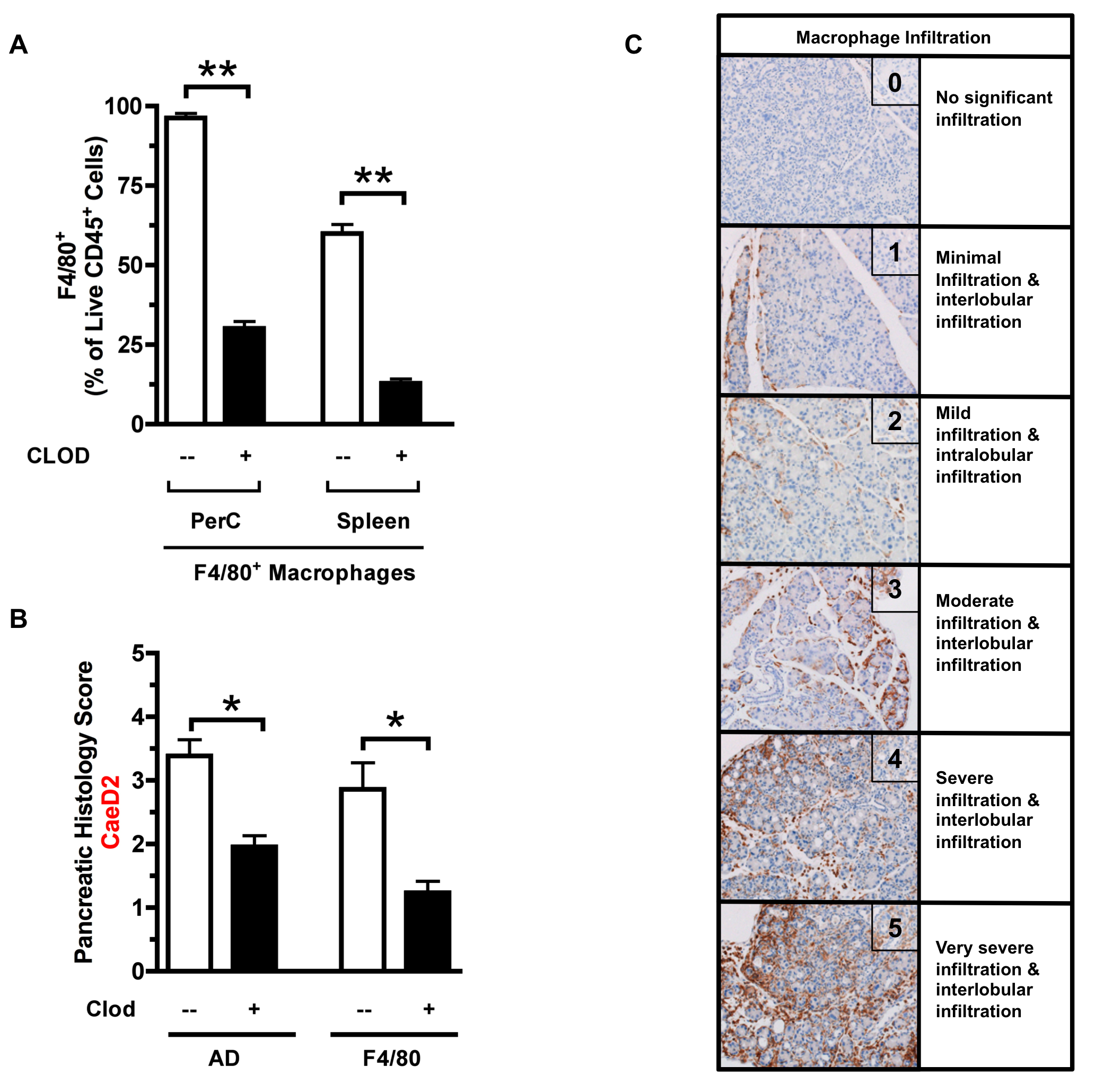

Supplement: Figure S4 — Macrophages depletion improves extent of caerulein-induced injury. (A) Percentage of F4/80+ macrophages present among CD45+ immune cells in the peritoneal cavity (PerC) and spleen from Rag1−/− mice treated with liposomes containing PBS or clodronate at CaeD2; n = 3 mice per group. (B) Pancreatic histology score evaluating the degree of acinar de-differentiation and F4/80+ infiltration (n = 3, *P<0.05, **P≤0.01, t test). (C) Representative images demonstrating scoring system used to evaluate the extent of F4/80+ infiltration. (TIF) [file pone.0102125.s004.tif]

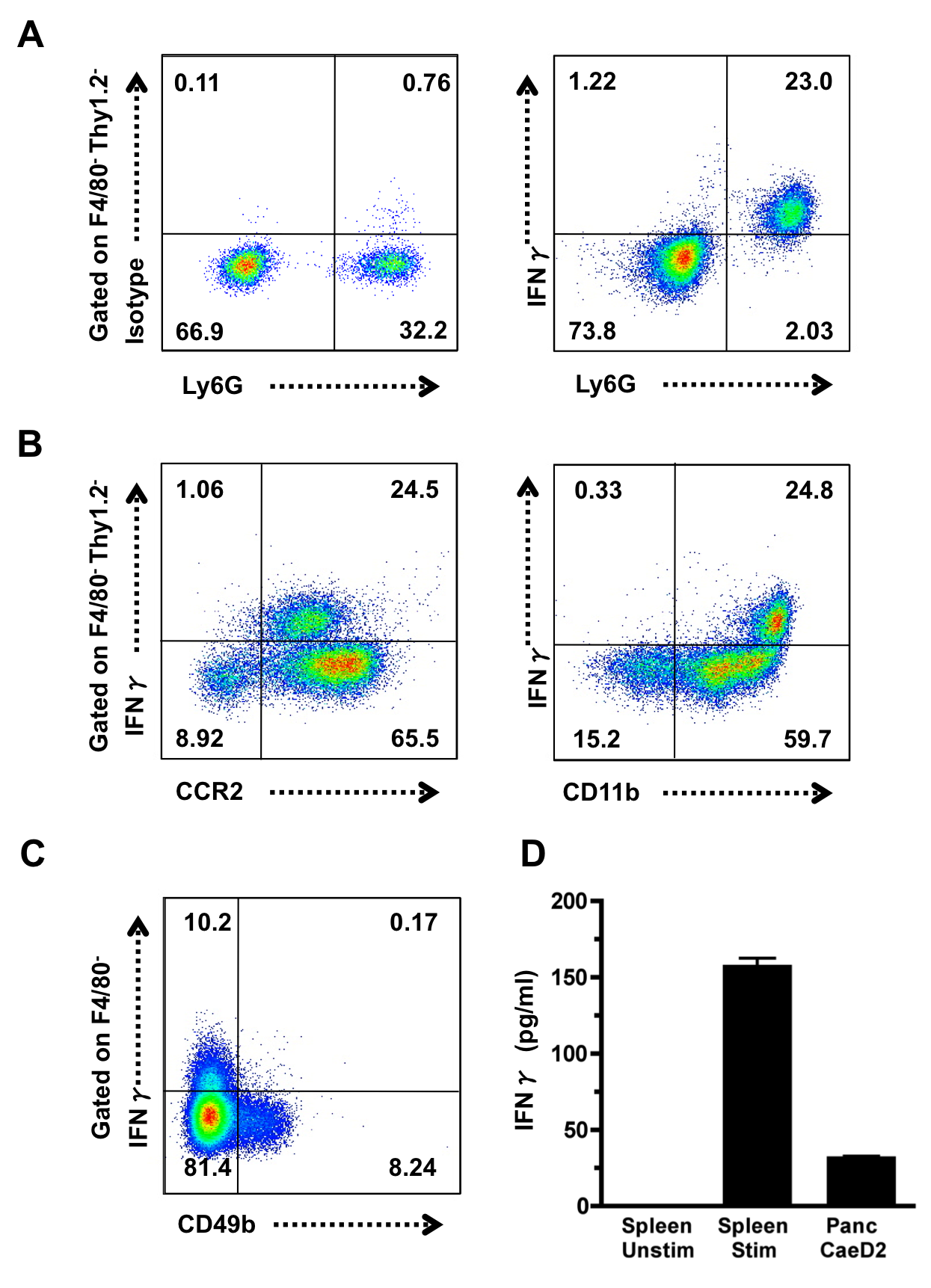

Supplement: Figure S5 — Phenotype of γPMNs. (A–C) Representative FACS analysis showing marker expression characterizing Ly6G+IFNγ+ cells. (A) Ly6G+IFNγ+ cells and isotype control (A), IFNγ+ cells are CCR2+ and CD11b+ (B), but not CD49b+ (C). (D) IFNγ levels present in media from cultured spleen cells +/− stimulation to release IFNγ and density fraction containing γPMNs isolated from the pancreas of WT mice at CaeD2 (n = 4). (TIF) [file pone.0102125.s005.tif]
